# Secondary Prevention of Suicide

**DOI:** 10.1371/journal.pmed.1000271

**Published:** 2010-06-01

**Authors:** Debora Ganz, M. Dolores Braquehais, Leo Sher

**Affiliations:** 1 Department of Psychiatry, Columbia University, New York, New York, United States of America; 2 New York State Psychiatric Institute, New York, New York, United States of America; 3 Department of Psychiatry, Vall d'Hebron University Hospital, Barcelona, Spain

## Abstract

Leo Sher and colleagues discuss recent research on interventions to prevent secondary suicide and discuss the additional research that is needed.

## The Need for Secondary Prevention of Suicide

Suicide poses major threats to public health worldwide. In 2002, suicide accounted for about 30,000 deaths in the US alone [1] and approximately 877,000 deaths worldwide—1.5% of the global burden of disease [2]. Suicide should and can be prevented. 83% of people who commit suicide have had contact with a primary care physician within a year of their death and up to 66% of people who commit suicide have had such contact within a month of their death [3].

Suicidal behavior has been conceptualized as a continuum of thoughts and behaviors ranging from suicidal ideation to completed suicide. Recent retrospective research delineates seven distinct categories of “suicidality”: (1) completed suicide, (2) suicide attempt, (3) preparatory acts toward imminent suicidal behavior, (4) suicidal ideation, (5) self-injurious behavior without intent to die, (6) nondeliberate self-harm, and (7) self-harm behavior with unknown suicidal intent [4].

Suicide prevention can be primary, secondary, or tertiary. Primary suicide prevention aims to reduce the number of new cases of suicide in the general population [5]. Secondary suicide prevention aims to decrease the likelihood of a suicide attempt in high-risk patients [5]. Tertiary suicide prevention occurs in response to completed suicides and attempts to diminish suicide contagion (clusters of suicides in a geographical area that occur predominantly among teenagers and young adults) and copy-cat suicides [5],[6].

Secondary suicide prevention is particularly important but not always given the attention that it deserves, in part because research into secondary prevention is only just starting to be applied to clinical practice. In this article, we discuss recent research on the evaluation of suicidal risk and on different kinds of secondary suicide prevention interventions that aim to reduce that risk. We also indicate how these interventions are currently being applied and what additional research is needed.

## Clinical Evaluation of Suicide Risk

Suicide is often difficult to predict due to its complex nature [7],[8]. Some of the risk factors that contribute to suicidal behavior are shown in Box 1 [1],[5],[9]–[11]. Research shows that these suicide risk factors are additive but can be divided into underlying causes such as biological and psychological factors, and more proximal stressors such as life events or a major depressive episode (Box 1) [7],[11]. Clinicians and others (termed gatekeepers by Mann et al. [12]) dealing with individuals who may be at risk for suicide should be taught to recognize, assess, and address such factors and to appropriately screen at-risk patients for suicidality.

**Box 1. Risk Factors for Suicide**

Biological risk factors for suicide include:

- Low cerebrospinal fluid 5-hydroxyindolacetic acid levels
- Hypothalamic-pituitary-adrenal axis dysregulation
- Low blood cholesterol levels
- Medical or neurological illnesses (such as multiple sclerosis, stroke, Huntington disease, and epilepsy)
- Cigarette smoking

Psychological risk factors include:

- Acceptability of suicide
- A childhood history of physical or sexual abuse
- Discouraged help-seeking behavior
- Aggressive/impulsive traits
- Pessimism
- Hopelessness
- Low self-esteem
- Poor access to psychiatric treatment

More proximal stressors that indicate an increased suicide risk include:

- Relationship problems
- Financial troubles
- A family or personal history of suicide
- Major depression
- Substance use

The clinical evaluation of the medical and psychiatric history of a patient and of their current state is the crucial and essential element of the suicide assessment process. This evaluation enables the clinician to identify risk factors and protective factors, to determine the patient's immediate safety and the best setting for treatment, and to develop a differential diagnosis and treatment strategies.

Psychiatric illness is a major contributing factor to suicide risk, with mood disorders such as major depressive disorder and bipolar disorder being associated with about 60% of suicides [12]–[14]. Indeed, psychiatric disorders are diagnosed in more than 90% of completed suicides, and more than 80% of these disorders are untreated at the time of death [13],[14]. Thus, the recognition and treatment of individuals with psychiatric disorders, specifically mood disorders, are essential components of secondary suicide prevention. In addition, the subjective rating of the severity of depression is one of the most powerful predictors of future suicidal acts [11]. Therefore, assessing and managing depression as well as being aware of the suicide risks in psychologically, medically, and neurologically disordered individuals is an important aspect of secondary suicide prevention [11]. Consequently, physicians need to be taught to recognize depression and to be educated about the association between mental disorders and suicide.

Additional information about the individual who may be at risk for suicide, such as depositions, medical and psychiatric treatment records, and toxicology screenings, should also be incorporated into the assessment. Additional information of this sort may be especially helpful, because information from individuals with mood disorders, borderline traits, or psychosis can be unreliable. Interviews with friends and relatives may also be helpful in assessing suicide risk.

Equally importantly, clinicians and other professionals in a position to offer help should not hesitate to ask patients about suicidal ideation because, while it may seem surprising, patients will often talk frankly about their suicidal thoughts and tendencies if given the opportunity. Failure to ask about suicidal ideation may be related to the care provider's discomfort with the topic, lack of time, or lack of skills in this area. Clinicians need to overcome these obstacles to provide appropriate care to their patients.

A final component of the clinical assessment of suicide risk is the gathering of information about current and past suicidal ideation and behavior, and about psychiatric conditions associated with suicidal behavior. There are many self-report and clinician-administered scales that measure different aspects of suicidal behavior or mental health conditions related to it (Box 2) [15]–[20]. Although these scales are reliable and have adequate concurrent validity, at present they are more useful as research tools than in the clinical setting, because they are limited in their assessment of comorbid risk factors.

**Box 2. Rating Scales for Suicide Behavior and Related Mental Health Conditions**

The most widely used scales for rating suicidal behaviors include:

- The Scale for Suicidal Ideation [15] has good reported reliability and validity and measures three major factors: active suicidal desire, specific plans for suicide, and passive suicidal desire.
- The Suicide Intent Scale [16] measures the degree of suicide intent.
- The Risk-Rescue Rating Scale [17] is an interviewer-administered measure that assesses the lethality and intent of a suicide attempt.
- The Columbia-Suicide Severity Rating Scale [4] assesses severity of suicidal ideation and tracks suicidal events.
- The Beck Hopelessness Scale [18] is a self-report inventory designed to measure three major aspects of hopelessness: feelings about the future, loss of motivation, and expectations.
- The Hamilton Depression Rating Scale [19] is a clinician-applied scale rating dimensions of depression.
- The Beck Depression Inventory [20] is a multiple-choice self-report inventory that measures the severity of depression.

## The Search for Biological Markers for Suicide

Many researchers have been trying to find biological markers related to suicidal behavior that could improve secondary suicide prevention. Several biological features related to failures in neurotransmitter and neuroendocrine systems, such as the serotonergic, noradrenergic, dopaminergic, and hypothalamic-pituitary-adrenocortical (HPA) systems, have been proposed [21],[22]. For example, considerable evidence accrued using various research approaches suggests a potentially causal association between suicidal behavior and the serotonin neurotransmission system [21]–[23]. Similarly, there is some evidence that dysregulation of HPA axis function may be involved in suicidal behavior in the context of acute stress response to life events [24],[25]. In particular, nonsuppression of the HPA axis by dexamethasone is associated with a 14-fold increase in the likelihood of suicide during 15 years of follow-up [25]. Finally, postmortem analyses of the noradrenergic system, which has been studied because it is involved in the regulation of the stress response, have revealed fewer noradrenergic neurons in the locus coeruleus, elevated brainstem levels of tyrosine hydroxylase, and reduced levels of postsynaptic adrenergic receptors in the cortex in people who commit suicide compared to the general population [24]. However, these findings may be related to an increased stress response before suicide rather than being a cause of suicide.

To date, the most promising biological predictors of suicidal behavior are low cerebrospinal fluid 5-hydroxyindoleacetic acid (5-HIAA, the main serotonin metabolite) and HPA axis dysregulation as indicated by dexamethasone nonsuppression [25],[26]. However, none of the putative biological markers identified to date are sensitive or precise enough to recommend their routine use in the clinical setting; additional translational and clinical studies are needed to understand the complex brain–mind relationship involved in suicidal behaviors.

## What Are the Most Effective Secondary Suicide Prevention Strategies?

In a recent systematic review of suicide prevention strategies, Mann et al. [12], found evidence of effectiveness in five secondary suicide prevention methods: pharmacological interventions, psychological interventions, follow-up care, reduced access to lethal means, and responsible media reporting of suicide [12].

Antidepressant medications are the most widely used pharmacological interventions in secondary suicide prevention, but studies of their effectiveness in reducing suicide attempts and completed suicides have had mixed results [12]. For example, although population studies show a decrease in suicide rates in the 27 countries with the greatest increase in selective serotonin reuptake inhibitor (SSRI) prescription [27], in 2003 and 2004 US and European regulators issued warnings about a possible association between antidepressant use and suicidal thinking and behavior. Since then, a meta-analysis [28] of randomized controlled trials has suggested that SSRIs may increase suicide ideation compared with placebo, but observational studies have suggested that SSRIs do not increase suicide risk any more than older antidepressants. If SSRIs do increase suicide risk in some patients, the number of additional deaths must be very small because ecological studies have generally found that suicide mortality has declined (or at least not increased) as SSRI use has increased. Moreover, Gibbons et al. [29] recently reported that, although SSRI prescriptions for children and adolescents decreased in both the US and The Netherlands after warnings were issued about a possible suicide risk with antidepressant use in pediatric patients, these decreases in SSRI use were associated with increases in suicide rates in children and adolescents.

A US Food and Drug Administration study recently reported that the risk of suicidality associated with use of antidepressants is age dependent [30]. Compared with placebo, the increased risk for suicidality and suicidal behavior among adults younger than 25 taking antidepressants approaches that observed in children and adolescents. The effect of antidepressants seems to be neutral on suicidal behavior but possibly protective for suicidal ideation in adults aged 25–64 and seems to reduce the risk of both suicidality and suicidal behavior in the elderly. Thus, the relation between antidepressants and suicidality needs further studies before this class of drugs (SSRIs in particular) can be safely used for the secondary prevention of suicide.

In terms of psychological interventions, suicidal patients often benefit from therapies that address the repetition of suicidal thoughts and behaviors, treatment adherence, and other factors commonly associated with suicidality [31]–[36]. Cognitive therapy decreases both suicidal ideation and the reattempt rate of past suicide attempters [31], and intensive care plus outreach and interpersonal psychotherapy decrease suicidal ideation [32]. In borderline personality disorder, which is associated with suicidality [37], dialectical behavioral therapy (which teaches patients how to reverse their negative thoughts and behaviors) and partial hospitalization in a psychoanalytically oriented facility improve treatment adherence and reduce suicidal behavior more than standard after-care [33]. Problem-solving therapy works to improve the mediating factors of suicidality; such as hopelessness and depression [32]. Better psychological and pharmacological treatment of depression and alcoholism, even in the absence of overt suicidal thoughts or behaviors, also appears to decrease suicide rates [5],[7],[10].

Follow-up care to maintain adherence to therapy (in particular, antidepressant use) after suicide attempts is also an effective approach to secondary suicide prevention [12]. Follow-up care can be provided by a case manager or by psychiatric hospitalization when appropriate [34]. Social factors that should be addressed in follow-up treatment include availability and willingness of supports within the family, within a facility, or by a support person designated to monitor the at-risk person. Support individuals who should be contacted about the suicide risk and follow-up arrangements include general practitioners, private psychiatrists, case managers, family, and friends. Regions that provide such follow-up care have lower treatment dropout rates and fewer repeat suicide attempts [36]. Some interventions, however, such as telephone and psychosocial follow-up, have shown no difference in reattempt rate and suicidal ideation when compared with standard after-care [12].

Many studies show that suicides by particular methods (for example, firearms, domestic gas, or pesticides) decrease after the introduction of legal restrictions that reduce access to such means [12]. This reduction in suicide rates is particularly influential in regions where the specific means restriction correlates with a common method of suicide [25],[26],[38],[39]. For example, in the UK the reduction of the mean percentage of carbon monoxide in domestic gas since 1958 and the reduced availability of analgesics since the mid-1990s have both decreased UK suicide rates [39]. Although method substitution is possible, such results show that the restriction of lethal means can save lives, mainly by decreasing the acute risk of suicide, which is related to impulsive suicidal behaviors.

Finally, many studies have exposed a need for a decrease in reporting of suicide and for responsible reporting. Media blackouts in reporting suicide have coincided with a decrease in suicide rates [40], possibly because reports of suicide in the media present suicide as a viable solution to life's problems and/or glamorize suicide for vulnerable individuals [41]. The Internet is also of increasing concern, with blogs and chat rooms socializing suicide and providing accessible instructions for suicide [42]. For these reasons, guidelines have recently been produced for the responsible reporting of suicide [12],[43]. However, the efficacy of these guidelines has not yet been assessed.

## The Future of Secondary Suicide Prevention

Despite our increasing knowledge about secondary suicide prevention, there are still many gaps in the research. The prescription of SSRIs and their impact on suicidal inclinations, especially in depressed children and adolescents, remain hotly debated topics [44]. Similarly, the most effective combinations of psychotherapeutic and pharmacologic interventions for suicidal patients have yet to be determined. And, while follow-up care has proven an effective element of suicide prevention, exactly which interventions are most effective remains unclear.

Nevertheless, much of what we have learnt about secondary suicide prevention through research can now be applied to the real world. For example, we know that to provide the best secondary suicide prevention, clinicians must learn how to evaluate at-risk individuals properly. We know that after completing these assessments, clinicians can now use well-researched psychological and pharmacological methods to decrease the levels of suicidality in their patients. We know that legally restricting access to lethal means and responsible media reporting of suicide correlate with a decrease in suicides worldwide [12]. And we know that the education of clinicians and society at large about suicide prevention is crucial.

Looking to the future, thorough evaluations and appropriate treatments of patients with depressive disorders and other psychiatric illnesses should help to improve the efficacy of secondary prevention of suicide. But, it is also clear that more research into new approaches for the prevention and treatment of suicidal behavior remains essential.
